# Draft genome sequence of *Leptospira yasudae* strain Ss3a1f from Estero De Paco, Manila, Philippines

**DOI:** 10.1128/mra.00547-26

**Published:** 2026-06-11

**Authors:** Ram Julius L. Marababol, Windell L. Rivera

**Affiliations:** 1 Pathogen-Host-Environment Interactions Research Laboratory, Institute of Biology, College of Science, University of the Philippines Diliman 172582, Quezon City, Philippines; 2 Philippine Genome Center, University of the Philippines System 54726, Quezon City, Philippines; DOE Joint Genome Institute, Berkeley, California, USA

**Keywords:** *Leptospira yasudae*, whole genome, Novaseq X, environmental sample, Estero de Paco

## Abstract

*Leptospira yasudae* is one of the pathogenic species of the genus *Leptospira*. Here, we report the draft genome sequence of *L. yasudae* strain ss3a1f from the Philippines isolated from an environmental soil sample collected in Estero de Paco, Manila.

## ANNOUNCEMENT

*Leptospira* is the causative agent of leptospirosis, a neglected tropical disease that is highly prevalent in the Philippines (1–4). The genus comprises more than 20 pathogenic, high-virulence species responsible for severe infections in humans and animals (5–8). One of these is *Leptospira yasudae*, which is frequently isolated from hosts but has also been reported as free-living in the environment, posing infection risks to both humans and animals (8–13).

A 20 g soil sample was collected from Estero de Paco (14.57985° N, 120.9934° E) at the end of the wet season on 13 March 2024. The sample was mixed with phosphate-buffered saline and shaken to obtain a homogeneous suspension. The mixture was then allowed to settle for 1 h, after which the supernatant was carefully collected without disturbing the settled particulates. The supernatant was incubated in the dark at 30°C for 2 weeks prior to inoculation into complete Ellinghausen–McCullough–Johnson–Harris (EMJH) medium, composed of base EMJH supplemented with 10% EMJH enrichment, 1% rabbit serum, and 0.01 mg/mL sodium pyruvate. The inoculated cultures were examined daily for 4 days using a phase-contrast microscope (Zeiss Axiolab 5) to detect the presence of spirochetes exhibiting helical characteristics, spiral-shaped morphology, with hooks on both ends, measuring approximately 0.1 µm in diameter and a length of 6–20 µm. Once spirochetes were confirmed, the cultures were transferred to semi-solid (0.4% agar) EMJH medium. Subsurface colonies were subsequently picked and further cultured in complete EMJH medium. A 2-week-old culture of putative *Leptospira* isolates showing visible Dinger’s ring formation was subjected to DNA extraction using the Monarch Spin DNA Gel Extraction Kit (NEB, USA), following the manufacturer’s instructions.

Sequencing libraries were prepared using the Illumina TruSeq Nano DNA Library Prep Kit with a 350-bp target insert size (Illumina, USA) and sequenced on a NovaSeq X platform using 150-bp paired-end reads (Illumina, USA). Sequencing adapters and low-quality reads were removed using Trimmomatic v0.36 using Q40 threshold (14), and read quality was assessed using FastQC v0.12.1 (15). Initial quality control yielded a total of 42,202,867 high-quality individual reads passing the set threshold. The coverage of high-quality reads was then assessed using CoverM.v0.7.0 (16). These high-quality reads were then assembled *de novo* using SPAdes v3.15.3 (17, 18). The resulting contigs were evaluated using QUAST v5.3.0 (19) and CheckM2 v1.1.0 (20) and subsequently annotated using Proksee (Build 2026-02-25) (21) with Bakta v1.8.2 (22) and FastANI v1.2.0 (23) for identity confirmation. All tools were run with default parameters unless otherwise specified.

The data set achieved a coverage depth of more than 250×. *De novo* assembly yielded a genome size of 4,503,556 bp distributed across 37 contigs, with an N50 of 384,449 bp, L50 of 3, and L90 of 14. CheckM2 analysis indicated a draft genome completeness of 99.92%. The genome has an overall GC content of 45.39% and contains 4,113 coding sequences, 38 transfer RNAs, 4 ribosomal RNAs, 1 transfer-messenger RNA, and 1 oriC. Average Nucleotide Identity analysis showed 98.96% similarity to *L. yasudae* F1 (GCF_003545925.1) and 99.02% similarity to *L. yasudae* L1S1 (GCF_004770135.1), both of which are isolated from a water sample in Mayotte and a soil sample from in Salvador, Brazil, respectively (9, 12).

The availability of this draft genome provides valuable insights into the environmental persistence of pathogenic *Leptospira*, which remains a significant but under-characterized public health concern in countries with limited genomic surveillance such as the Philippines.

## Data Availability

This whole-genome project is deposited in the National Center for Biotechnology Information (NCBI) GenBank database under accession number JBYAXG000000000. The BioProject accession number is PRJNA1459639, and the BioSample accession number is SAMN57560757. The Illumina NovaseqX raw data have been deposited in the NCBI Sequence Read Archive under accession number SRR38327166.

## References

[1] Rajapakse S, Fernando N, Dreyfus A, Smith C, Rodrigo C. 2025. Leptospirosis. Nat Rev Dis Primers 11:32. doi:10.1038/s41572-025-00614-5 40316520

[2] Villanueva SYAM, Ezoe H, Baterna RA, Yanagihara Y, Muto M, Koizumi N, Fukui T, Okamoto Y, Masuzawa T, Cavinta LL, Gloriani NG, Yoshida S. 2010. Serologic and molecular studies of Leptospira and leptospirosis among rats in the Philippines. Am J Trop Med Hyg 82:889–898. doi:10.4269/ajtmh.2010.09-0711 20439972 PMC2861393

[3] Interior JS, Bigay-Iringan KJJ, Bondad-Delson RND, Iringan RAA, Calderon XMS, Castro AGP. 2025. Leptospirosis in the Philippines: confronting the structural roots of a recurring threat. Pathogens 14:963. doi:10.3390/pathogens14100963 41156574 PMC12567350

[4] Almazar CA, Montala YB, Rivera WL. 2026. Leptospirosis in Southeast Asia: investigating seroprevalence, transmission patterns, and diagnostic challenges. Trop Med Infect Dis 11:18. doi:10.3390/tropicalmed11010018 41591227 PMC12846544

[5] Bilung LM, Tahar AS, Pui CF, Bakeri MKS, Su’ut L, Ngui R, Kira R, Apun K. 2026. Leptospira and Leptospirosis: a review of species classifications, genomes, morphological structures, antimicrobial resistances, transmissions, and clinical manifestations. Curr Microbiol 83:122. doi:10.1007/s00284-026-04722-7 41489657 PMC12769562

[6] Helman SK, Tokuyama AFN, Mummah RO, Stone NE, Gamble MW, Snedden CE, Borremans B, Gomez ACR, Cox C, Nussbaum J, Tweedt I, Haake DA, Galloway RL, Monzón J, Riley SPD, Sikich JA, Brown J, Friscia A, Sahl JW, Wagner DM, Lynch JW, Prager KC, Lloyd-Smith JO. 2023. Pathogenic Leptospira are widespread in the urban wildlife of southern California. Sci Rep 13:14368. doi:10.1038/s41598-023-40322-2 37658075 PMC10474285

[7] Sayanthi Y, Susanna D. 2024. Pathogenic Leptospira contamination in the environment: a systematic review. Infect Ecol Epidemiol 14:2324820. doi:10.1080/20008686.2024.2324820 38511199 PMC10953783

[8] Giraud-Gatineau A, Nieves C, Harrison LB, Benaroudj N, Veyrier FJ, Picardeau M. 2024. Evolutionary insights into the emergence of virulent Leptospira spirochetes. PLoS Pathog 20:e1012161. doi:10.1371/journal.ppat.1012161 39018329 PMC11285912

[9] Casanovas-Massana A, Hamond C, Santos LA, de Oliveira D, Hacker KP, Balassiano I, Costa F, Medeiros MA, Reis MG, Ko AI, Wunder EA. 2020. Leptospira yasudae sp. nov. and Leptospira stimsonii sp. nov., two new species of the pathogenic group isolated from environmental sources. Int J Syst Evol Microbiol 70:1450–1456. doi:10.1099/ijsem.0.003480 31184568 PMC10197099

[10] Shamsusah NA, Agustar HK, Amran F, Hod R. 2021. Draft genome sequence of Leptospira yasudae strain BJ3, isolated from the soil of an ex situ wild animal conservation area. Microbiol Resour Announc 10:e0072321. doi:10.1128/MRA.00723-21 34591676 PMC8483709

[11] Casanovas-Massana A, Vincent AT, Bourhy P, Neela VK, Veyrier FJ, Picardeau M, Wunder Jr. EA. 2019. Leptospira dzianensis and Leptospira putramalaysiae are later heterotypic synonyms of Leptospira yasudae and Leptospira stimsonii. Int J Syst Evol Microbiol 71:004713. doi:10.1099/ijsem.0.004713 33620308 PMC8375420

[12] Vincent AT, Schiettekatte O, Goarant C, Neela VK, Bernet E, Thibeaux R, Ismail N, Mohd Khalid MKN, Amran F, Masuzawa T, Nakao R, Amara Korba A, Bourhy P, Veyrier FJ, Picardeau M. 2019. Revisiting the taxonomy and evolution of pathogenicity of the genus Leptospira through the prism of genomics. PLoS Negl Trop Dis 13:e0007270. doi:10.1371/journal.pntd.0007270 31120895 PMC6532842

[13] Thongdee M, Chaiwattanarungruengpaisan S, Paungpin W, Sungpradit S, Jiemtaweeboon S, Tiyanun E, Ruchisereekul K, Suwanpakdee S, Thaipadungpanit J. 2026. Pathogenic Leptospira species identified in dogs and cats during neutering in Thailand. PLoS Negl Trop Dis 20:e0013421. doi:10.1371/journal.pntd.0013421 41637464 PMC12871963

[14] Bolger AM, Lohse M, Usadel B. 2014. Trimmomatic: a flexible trimmer for Illumina sequence data. Bioinformatics 30:2114–2120. doi:10.1093/bioinformatics/btu170 24695404 PMC4103590

[15] Andrews S. 2026. FastQC a quality control tool for high throughput sequence data. https://www.bioinformatics.babraham.ac.uk/projects/fastqc.

[16] Aroney STN, Newell RJP, Nissen JN, Camargo AP, Tyson GW, Woodcroft BJ. 2025. CoverM: read alignment statistics for metagenomics. Bioinformatics 41:btaf147. doi:10.1093/bioinformatics/btaf147 40193404 PMC11993303

[17] Antipov D, Korobeynikov A, McLean JS, Pevzner PA. 2016. hybridSPAdes: an algorithm for hybrid assembly of short and long reads. Bioinformatics 32:1009–1015. doi:10.1093/bioinformatics/btv688 26589280 PMC4907386

[18] Nurk S, Bankevich A, Antipov D, Gurevich A, Korobeynikov A, Lapidus A, Prjibelsky A, Pyshkin A, Sirotkin A, Sirotkin Y, Stepanauskas R, McLean J, Lasken R, Clingenpeel SR, Woyke T, Tesler G, Alekseyev MA, Pevzner PA. 2013. Assembling genomes and mini-metagenomes from highly chimeric reads, p 158–170. *In* Deng M, Jiang R, Sun F, Zhang X (ed), Research in computational molecular biology. Springer, Berlin, Heidelberg. 10.1089/cmb.2013.0084 PMC3791033 24093227

[19] Gurevich A, Saveliev V, Vyahhi N, Tesler G. 2013. QUAST: quality assessment tool for genome assemblies. Bioinformatics 29:1072–1075. doi:10.1093/bioinformatics/btt086 23422339 PMC3624806

[20] Chklovski A, Parks DH, Woodcroft BJ, Tyson GW. 2023. CheckM2: a rapid, scalable and accurate tool for assessing microbial genome quality using machine learning. Nat Methods 20:1203–1212. doi:10.1038/s41592-023-01940-w 37500759

[21] Grant JR, Enns E, Marinier E, Mandal A, Herman EK, Chen C, Graham M, Van Domselaar G, Stothard P. 2023. Proksee: in-depth characterization and visualization of bacterial genomes. Nucleic Acids Res 51:W484–W492. doi:10.1093/nar/gkad326 37140037 PMC10320063

[22] Schwengers O, Jelonek L, Dieckmann MA, Beyvers S, Blom J, Goesmann A. 2021. Bakta: rapid and standardized annotation of bacterial genomes via alignment-free sequence identification. Microb Genom 7:000685. doi:10.1099/mgen.0.000685 34739369 PMC8743544

[23] Jain C, Rodriguez-R LM, Phillippy AM, Konstantinidis KT, Aluru S. 2018. High throughput ANI analysis of 90K prokaryotic genomes reveals clear species boundaries. Nat Commun 9:5114. doi:10.1038/s41467-018-07641-9 30504855 PMC6269478

